# Global marine biosecurity and ship lay-ups: intensifying effects of trade disruptions

**DOI:** 10.1007/s10530-022-02870-y

**Published:** 2022-07-14

**Authors:** Gregory M. Ruiz, Bella S. Galil, Ian C. Davidson, Sarah C. Donelan, A. Whitman Miller, Mark S. Minton, Jim R. Muirhead, Henn Ojaveer, Mario N. Tamburri, James T. Carlton

**Affiliations:** 1 Smithsonian Environmental Research Center, Edgewater, MD USA; 2 The Steinhardt Museum of Natural History and Israel National Center for Biodiversity Studies, Tel Aviv University, Tel Aviv, Israel; 3 Cawthron Institute, Nelson, New Zealand; 4 Pärnu College, University of Tartu, Pärnu, Estonia; 5 National Institute of Aquatic Resources, Technical University of Denmark, Lyngby, Denmark; 6 Chesapeake Biological Laboratory, University of Maryland Center for Environmental Science, Solomons, Maryland, USA; 7 Williams College-Mystic Seaport Ocean and Coastal Studies Program, Mystic, CT USA

**Keywords:** Biofouling, Biosecurity, COVID-19, Trade disruptions, Shipping, Suez Canal

## Abstract

Recent global trade disruptions, due to blockage of the Suez Canal and cascading effects of COVID-19, have altered the movement patterns of commercial ships and may increase worldwide invasions of marine non-indigenous species. Organisms settle on the hulls and underwater surfaces of vessels and can accumulate rapidly, especially when vessels remain stationary during lay-ups and delays. Once present, organisms can persist on vessels for long-periods (months to years), with the potential to release propagules and seed invasions as ships visit ports across the global transportation network. Shipborne propagules also may be released in increasing numbers during extended vessel residence times at port or anchor. Thus, the large scale of shipping disruptions, impacting thousands of vessels and geographic locations and still on-going for over two years, may elevate invasion rates in coastal ecosystems in the absence of policy and management efforts to prevent this outcome. Concerted international and national biosecurity actions, mobilizing existing frameworks and tools with due diligence, are urgently needed to address a critical gap and abate the associated invasion risks.

## Introduction

**Fig. 1:**
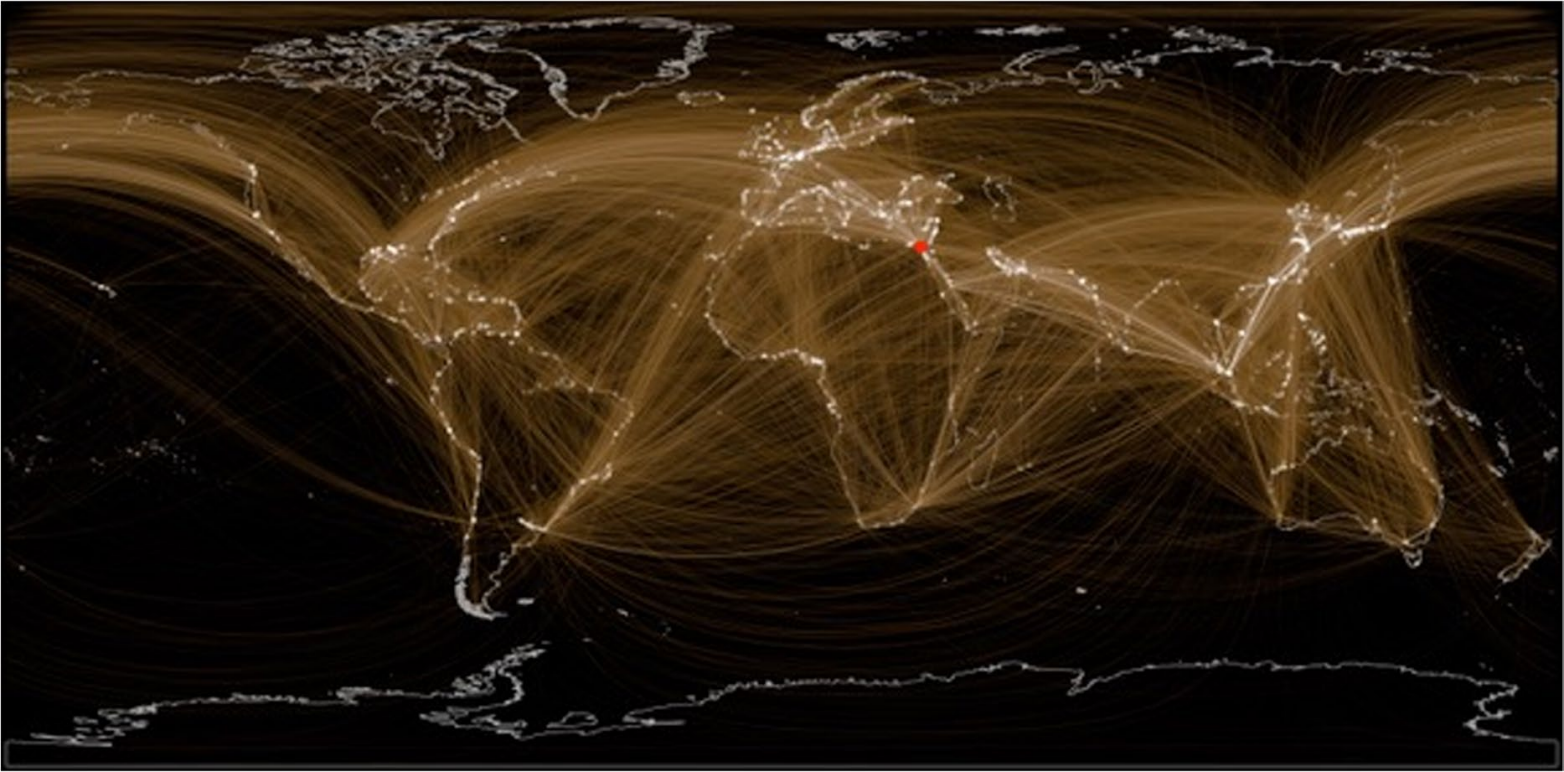
The global network of port callings for commercial vessels that transited the Suez Canal in 2018. Shown is the distribution of ports visited by vessels that transited the Suez (shown in red) from January–December 2018, including the 45 days before and after. Port brightness is proportional to number of visits during this period (totals: 346,501 separate port callings, 3889 unique ports and anchorages, 6693 unique vessels). Data provided by S & P Global

On the morning of March 23, 2021, the 400 m-long “Ever Given”, one of the largest container ships in the world, became wedged across the Suez Canal, bringing maritime traffic to a halt for six days in the world's busiest inter-ocean passage. A shipping superhighway, used by > 18,000 commercial ship transits per year, the canal links a vast constellation of ports around the world (Fig. 1). As the world watched, more than 350 commercial ships queued in the Red Sea, the Mediterranean, and in the canal itself (Lind et al. 2021). The Suez Canal reopened on March 29, yet on April 3 the queue waiting to transit still numbered around 200 vessels. Suez-delayed ships caused capacity bottlenecks and backlogs at far away destination ports: as port authorities scurried to allot scarce berthing slots and storage space, large numbers of vessels were queueing at anchorages the world over (Kickham 2021).

**Fig. 2:**
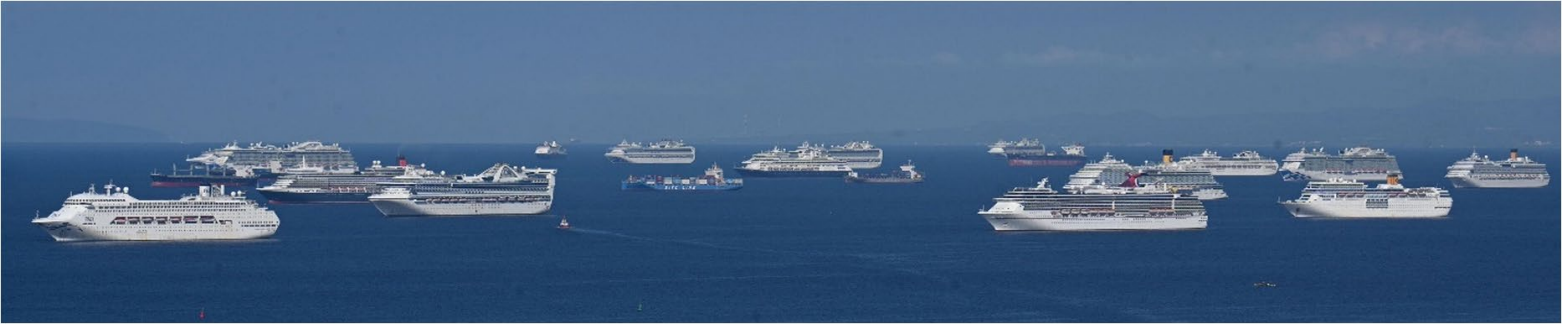
Cruise ships aggregated at anchor at Manila Bay, Philippines (May 31, 2020), in response to COVID-19 disruptions and restrictions. (Photo by Ted Aljibe/AFP via Getty Images)

The Suez Canal blockage overlapped with the COVID-19 pandemic crisis, which has been disrupting global shipping patterns since early 2020. Cruise ships have been among the hardest hit maritime transportation sectors (March et al. 2021; Millefiori et al. 2021). With no customers and thus nowhere to go, many cruise ships were laid up forming stationary aggregations nearshore (Fig. 2), mostly in tropical and sub-tropical seas (Rogoway 2021). Containerships have also experienced extended delays at ports around the world, due to supply-chain interruptions, resulting from a surge in demands for goods, a shortage of shipping containers, and reduced dockside capacity (Berger 2021; Goodman 2021; Varley and Murray 2021).

While pandemic impacts on cruise ships began almost immediately, those on containerships built more gradually, causing backups and delays that grew more acute throughout 2021. For example, high COVID-19 infection and exposure rates among transportation and logistics personnel caused delays at major North American Pacific Coast ports that were exacerbated by a surge of incoming trans-Pacific cargo to supply booming consumer spending on goods in the U.S. This combination produced extensive harbor congestion and knock-on supply chain disruptions, especially for containerships (Berger 2021; Goodman 2021). Recently, over 100 anchored ships were waiting to berth at the ports of Los Angeles and Long Beach in a queue stretching 20 km, in contrast to the few ships normally at anchor there (Berger 2022). Shipping officials projected the backups will continue into 2023 (Saraiva and Murray 2021; Berger 2022).

## Ships laid-up together may increase global marine bioinvasions

Beyond the much-discussed and high-profile economic disruptions, ships’ protracted standstills and shifts in operational tempo may have widespread environmental impacts. Foremost among these are marine biological invasions, resulting from the ship-mediated transfer of species from one global location to another. Such species transfers enable the establishment and spread of non-indigenous species (NIS) to new regions that threaten economies, health, biodiversity, cultural uses, and ecosystem function, with potentially large and lasting impacts (Ruiz et al. 2015; Bailey et al. 2020).

Stationary ships integrate several key processes known to increase the likelihood of invasions. It is well known that ships transfer NIS as biofouling organisms accumulating on their hulls and other exposed underwater surfaces, as well as planktonic and nektonic organisms entrained in ballast tanks, and together these ship-mediated transfers drive global invasion dynamics in coastal ecosystems (Bailey et al. 2020). Importantly, when vessels remain immobile for extended periods, biotic exchange between ship and environment (and among closely clustered ships) can increase, with more time and opportunity for organisms to colonize vessels (Davidson et al. 2020) or jump ship to surrounding waters via reproduction, mobility, or fragmentation (Apte et al. 2000; Minchin and Gollasch 2003). Thus, residence time is recognized as a key factor in biofouling accumulation on ships and subsequent invasion risk (Floerl and Coutts 2009; Davidson et al. 2016, 2020).

Ship lay-ups and prolonged queuing may increase chances of “super-spreader” events, where vessels accumulate heavy biofouling and transfer NIS at unusually high rates to subsequent downstream ports, both near and far (Fig. 1). Blockages and bottlenecks occurring in multiple regions expand the range of environmental conditions involved and the diversity (cumulative species pool) of biofouling organisms available to colonize vessels, thereby increasing the potential opportunities for species transfer events by vessels moving among suitably matched source and destination conditions. Although the likelihood of transfer and subsequent invasions of NIS may be relatively low during normal operations for any single vessel or voyage, and depends on multiple factors (Ruiz et al. 2000; Davidson et al. 2018), transport and invasion potential undoubtedly increases with the growing number of vessels and global regions exposed to layup events.

While biofouling can impose significant costs on vessel performance, such as increased fuel consumption (due to drag), that provide some incentives for its management, international policy to reduce associated NIS invasions is still emerging (Davidson et al. 2016; Tamburri et al. 2021). Currently, only voluntary biofouling guidelines exist for vessels operating globally (International Maritime Organization 2011) to reduce NIS invasions, with the exception of a few regions (Davidson et al. 2016; Tamburri et al. 2021).

The economic impact of laid-up and idled vessels (to ship operators), and urgency to return to business, may also constrain the effective use of in-water cleaning or other mitigation measures to reduce biofouling prior to re-entry to service that has now begun (Hines 2021). Since dry-dock hull-cleaning occurs at approximately 3–10 year intervals, biofouling accumulated in lay-up events can persist on vessels for years and may also facilitate further colonization, whereby species can recruit to existing biofouling and avoid the biocidal effects of antifouling coatings.

Thus, the cumulative effect of these multiple overlapping lay-up events has the potential to greatly increase invasion rates across the globe, although this increase may take years to detect, due to both lag-time in detection and limited marine surveillance efforts (Ruiz et al. 2000; Bailey et al. 2020). Instead of a short-term spike, these events may cause a more sustained invasion wave, as NIS establish and spread through the complex world-wide transportation network. The consequences of these current events for invasion dynamics and sustained impacts have received little attention, and to our knowledge, there is no plan to evaluate or mitigate associated invasion risks, outside of already existing practices.

## Biosecurity measures for vessel biofouling

Commercial ships have an average underwater exposed surface area of 1000s of square meters—a staggering 500,000,000 m^2^ across the global fleet—that is susceptible to biofouling (Moser et al. 2016). In 2011, the International Maritime Organization (IMO) adopted its voluntary “Guidelines for the control and management of ships' biofouling to minimize the transfer of invasive aquatic species” (International Maritime Organization 2011), and has recently agreed to review these and consider adding requirements (International Maritime Organization 2018). This follows a similar approach initiated over 30 years ago by IMO for treating ships’ ballast water, beginning with voluntary guidelines and transitioning to mandatory treatment to reduce NIS transfers (Albert et al. 2013; Scianni et al. 2021), with full implementation still years away.

There is an urgent need and complementary roles for both international and national biosecurity policy actions to limit these risks. Accelerated efforts by the IMO to review and revise the Biofouling Guidelines are critically needed to establish a protective and coordinated international regulatory biosecurity framework. These efforts should include regulations that drive explicit and required management responses for lay-up events, and incorporate reporting, inspection, and biofouling management plans to meet specific standards. While lay-ups and extended queuing are unexpected disruptions to normal operations, such disruptions will continue in response to economic down-turns (or upturns) and numerous other global events. Biosecurity measures must both anticipate and respond to these events and specifically to the associated high-risk for marine NIS transfer and invasions.

Although momentum is building for such a multilateral framework, this is likely still years away from all-important implementation of mandatory biofouling management. At present, New Zealand is unique in enforcing comprehensive national biofouling management on international vessels (Ministry for Primary Industries 2020; Georgiades et al. 2020). If more countries advance similar requirements, especially those that are key nodes in the trade network, their reach would extend far beyond national boundaries to reduce biofouling transfers across major international routes and ports. Such national efforts have enormous potential to dampen broadscale environmental, economic, and socio-cultural costs of novel biofouling invasions. Management actions already available for implementation include both underwater inspections of stationary vessels and in-water treatment and cleaning technologies to remove biofouling (Scianni and Georgiades 2019; Tamburri et al. 2020; Tamburri et al. 2021; Scianni et al. 2021). Tools exist or are emerging—the time has come to mobilize them effectively, to stem invasion risks associated with the current lay-ups and beyond.

## Data availability statement

The data that support the findings presented in Fig. 1 of this study are available from S & P Global but restrictions apply to the availability of these data, which were used under license for the current study, and so are not publicly available. Data are however available from the authors upon reasonable request and with permission of S & P Global.
